# Beverage Consumption Habits around the World: The Burden of Disease Attributable to Hydration

**DOI:** 10.3390/nu8110738

**Published:** 2016-11-18

**Authors:** Lluis Serra-Majem, Mariela Nissensohn

**Affiliations:** Research Institute of Biomedical and Health Sciences, University of Las Palmas de Gran Canaria, 35016 Las Palmas, Spain; mnissensohn@acciones.ulpgc.es

Dehydration occurs when the body loses more water than is taken in. It is often accompanied by disturbances in the body’s mineral salt or electrolyte balance, especially in the concentrations of sodium and potassium.

Populations at particular risk of hypohydration are the very young, those engaged in professions where fluid homeostasis is regularly challenged and the elderly. Limited data are available on the prevalence of hypohydration, but there is evidence to suggest that this may be relatively common among the western elderly populations [1]. The percentage of population with inadequate intake of water may vary from 5% to 35% in the different European countries [2].

The burden of disease from lack of access to clean water, poor sanitation and inadequate personal and food hygiene is well known, but consequences of inadequate water intake worldwide are far from being well understood. Recent research [3] into the risk of disease (bowel, metabolic and kidney diseases), disability (cognitive function, physical performance, headache, falls and accidents) and death is confirming the importance of poor hydration to overall disease burden and quality of life in a worldwide perspective.

Moreover, the number of hospitalizations for dehydration has steadily increased in recent decades. In this case, dehydration increases the healthcare burden in a direct way, as a disease itself. However, sometimes, dehydration appears as a comorbidity condition in a number of diseases. Dehydration has been defined as the second most common comorbidity, occurring in 14% of all hospitalizations [4]. In addition to its individual clinical impact; dehydration also represents an important public health issue by imposing a significant economic burden. Depending on the degree or magnitude of the dehydration in hospitalized patients, costs may increase by 7% to 8.5% [5]. Higher cost will be associated with an increase in hospital mortality, as well as with an increase in the utilization of intense short and long term care facilities, readmission rates and hospital resources, especially among those with moderate to severe hypernatraemia. Dehydration represents a potential target for intervention to reduce healthcare expenditures and improve patients’ quality of life [6]. However, the higher cost of dehydration is not only in hospitalized patients. Dehydration is one of the major factors limiting athletic performance and even mild dehydration can have a significant impact on working capacity and productivity [7]. Some studies have suggested a possible link between dehydration and the rise in industrial accidents during the summer, when workers are presumably thirstier [8]. A study published in 2004 found it had a real impact on performance in physical fields such as forestry, with slightly dehydrated workers being about 12% less productive than their fully hydrated peers [9].

Improving drinking habits during working and leisure time and developing comprehensive hydration guidelines for healthcare professionals and patients would be a cost-effective means of addressing the burden of hypohydration in developed countries.

Given the extent of the problem and its under-acknowledgment, will governments engage in research and awareness-raising strategy on the burden of hypohydration in the different countries and regions? Will governments address the burden of dehydration in the elderly as part of its action plan on active ageing to be proposed in the near future?

On the other side, a rising number of studies assert that sugar-containing drinks may play a key role in the etiology of overweight and obesity in children and adults [10]. However, whether this association is causa still remains controversial [11,12]. Public policy initiatives to reduce sugar in beverages consumption are the subject of current debate and different initiatives are actually developed worldwide. These should consider both, promoting an adequate water intake and a reduction in sugar and energy intake from beverages, across the different age and socioeconomic groups, all within the broader context of improving overall diet quality.

At this stage, the current Issue has received a great welcome from the scientific community which has responded to the proposal with huge interest. However, although the number of the papers published in the present supplement is really impressive (around 25), the needs for these kinds of studies have not decreased. The studies included in this supplement were developed in different settings and different populations around the world; the diversity methodologies employed in the quantitative assessment of beverages consumption and all the details that the results of the studies have shown may eventually help to adequately address their policies worldwide. Broadly, the papers in the present volume provide a valuable milestone on our journey to understand the impacts of the hydration on health and disease and they will be helpful for those planning future studies. Our main interest has been to gradually increase the general interest placed on this emerging and fascinating area of study.

As we move forward, our progress will continue to depend heavily on beverage intake assessment methods, and underpinning this is the need to further refine and document the validity of our methods. Again, those of us engaged in nutrition research will be highly appreciative of the great efforts of our colleagues who have produced this volume documenting our progress to date.
